# Army Reports

**Published:** 1862-09

**Authors:** H. Wardner

**Affiliations:** Brigade-Surgeon


ARTICLE XXVII.

### ARMY REPORTS.

FROM H. WARDNER, M.D., Brigade-Surgeon.

CORINTH, MISSISSIPPI, *Sept.* 9, 1862.

PROF. N. S. DAVIS.

DEAR SIR: — I send you herewith a copy of the remarks accompanying the reports of two of the surgeons of our division. Together they give a very clear idea of the condition and treatment of the men generally in this division.

The following is taken from the summary of my consolidated monthly report for August, 1862.

Mean strength, (*i. e.*, those present,) 7189.

Number treated, 1703, (this includes all diseases, both chronic and acute, for the month.

The daily average on the sick list is about 600.

Deaths during the month, 23.

Remarks accompanying the Monthly Report of Surgeon H. C. FOOTE:—

CAMP 22D OHIO VOL. INF., NEAR CORINTH, MISS., *August* 31, 1862.

There have been in the regiment scarce any cases of fever of a grave character, almost all have been tertian intermittents, and all yielding readily and permanently to quinine.

The camp is situated on elevated ground, which is dry and airy, but has been in use for that purpose for several months. A little west of north, and about 500 yards distant, the ground is low, flat, and damp, but not swampy.

The supply of water is abundant, but not very good, consisting too much of surface water.

The prevailing diseases have been diarrhœa and dysentery, often very obstinate, and in some cases terminating in ulceration of the bowels, in spite of every effort.

Various plans of treatment have been resorted to, all successful in mild cases; all ineffectual occasionally. Most cases of diarrhœa were readily controlled by mild mercurials, not pushed to ptyalism, followed by astringents and opium. The saline treatment proved efficacious in many cases of both diseases, but on the whole has not accomplished as much good as I had hoped. Some obstinate cases of diarrhœa and dysentery, which resisted other treatment, have been much benefited by the administration of Fowler's solution and laudanum.

Nitrate of silver and sulphate of copper have hitherto failed in accomplishing any good results, and, in some cases, both of diarrhœa and dysentery, undoubtedly aggravated the disease.

There has been a deficiency in the supply of medicines, until within a very short time, and I have, in consequence, been prevented from following the course of treatment that seemed most proper in many cases; and, also, debarred from full observation and comparison of different plans of treatment.

The rations of the men have been abundant, of good quality, and sufficiently varied; but the cookery has been, and is, as a general rule, abominable. The frying-pan has been the chief dependance, and the soups, as a general rule, are greasy and ill-flavored. The men pay but little attention to the advice or remonstrance of the surgeon on these points, and it is only when the company officers second his efforts that any good result is accomplished, and this happens so seldom that the general health of the regiment is not affected at all. It is, however, proper to state that cases of diarrhœa and dysentery have occurred in patients in the hospital, under treatment for other diseases, and whose diet was carefully regulated.

There have been no cases of scurvy, pure and simple, during this month, and scarce any since the regiment has been in service, but very many of the patients have suffered in a way that could not be accounted for, except on the supposition of some scorbutic taint. In various instances, but generally in cases of diarrhœa and dysentery, there has been marked œdema of the lower extremities, and sometimes of the face also. Partial paralysis of one or more limbs has not been uncommon, and in one or two cases, which proved fatal, there was ecchymosis, in one case quite extensive.

The treatment most successful with these cases, after subduing the active disease, was the administration of iron and acids, and nourishing diet.

There have been but few cases of disease of the respiratory organs, and these not severe.

The regiment has had a sufficient good supply of good tents, has not been on the march, and the weather has been favorable. There have been occasional showers but not long-continued rain, and no intensely hot weather.

Owing to want of instruments, I cannot give variations of temperature, etc. Very respectfully,

H. E. FOOTE,

Surgeon 22d Reg't. Ohio Vol. Inf.

Remarks accompanying the Monthly Report of Surgeon C. B. LAKE, 7th Iowa:—

SURGERY.—None in regiment.

FEVER, Continued or Typhoid.—None.

REMITTENT—Type mild—Symptoms usual.—*Treatment.*—A free purge of Sulph. M. G. or Hyd. Sub. Mur. and Castor Oil, followed by two or three 5 gr. doses Sulph. Quinina, and the case is arrested. Sulph. Cinchonia and Brandy are used as a tonic, if any after treatment is required.

INTERMITTENT—No unusual symptoms.—*Treatment.*—From 8 to 10 grs. Sulph. Quinina at one dose, as soon as the sweating stage is established, with 6 to 8 grs. pill Hyd., and that followed next day by a purgative, if the secretions are not active.

DIARRHŒA AND DYSENTERIA—Grade active.—*Cause.*—The eating of green fruits, pies, and other indigestible substances.

*Treatment.*—Thoroughly evacuant, at first followed by Anodyne and sometimes full doses of Sulph. Quinina. A common prescription with me, is:

℞.—Sulph. M. G. .................................... ℥i.

Tart. Ant. et Potas, ........................ ½ to 1 gr.

Mix and give. Repeated next day or day after, if necessary, followed by 5 gr. doses Pulvis. Doveri, if there is pain of the bowels. Wet cupping when there is tenderness of abdomen; perfect quiet; abstinence from all cold drinks; no food, except rice water or gruel. Under this treatment, it is altogether the exception if a case becomes chronic.

Chronic Diarrhœa and Dysenteria.—Chronic fluxes of the bowels have been the scourge of this regiment, and they are found in all forms and stages of the disease. The cause, I believe to have been the use of astringents and opiates, in the treatment of the acute disease. The treatment I have found most successful, since I came to the regiment, (which was last April,) is careful diet, dry cupping, counterirritation over the abdomen, and the exhibition of from 3 to 5 drops of Fowler's solution, two or three times daily. Under this treatment a large per cent. of the cases have entirely recovered.

Respiratory Diseases—Character mild—Symptoms usual.

*Treatment.*—Moderately antiphlogistic and expectorant, with quinina if showing miasmatic symptoms.

Rations.—The ration consists of flour, made into light yeast bread, and baked in adoba ovens; rice, bacon, and pickled pork two-thirds and fresh beef one-third of the time,—the beef being made mostly into soup; potatoes and onions whenever they can be procured; coffee.

The troops are sheltered with tents, of which there is a scant supply, except for the dry season. The weather is mostly dry. Wind South most of the time, occasionally changing to N.W.

Location of Camp.—The camp is located on dry table land, on the south side of a creek, fronting to the South. Sinks in the rear of regiment are filled every morning with fresh earth. Camp thoroughly policed. Tents struck frequently, and ground swept. Men all made to build bunks of polls or boards, about 18 inches from the ground, on which they sleep comfortably.

